# Evaluating the Potential of *Portulaca oleracea* L. for Parkinson's Disease Treatment Using a *Drosophila* Model with *dUCH*-Knockdown

**DOI:** 10.1155/2019/1818259

**Published:** 2019-04-18

**Authors:** Huynh Kim Thoa Truong, Man Anh Huynh, My Dung Vuu, Thi Phuong Thao Dang

**Affiliations:** ^1^Department of Molecular and Environmental Biotechnology, Faculty of Biology and Biotechnology, University of Science, Vietnam National University-Ho Chi Minh City, Ho Chi Minh City 700000, Vietnam; ^2^Laboratory of Molecular Biotechnology, University of Science, Vietnam National University-Ho Chi Minh City, Ho Chi Minh City 700000, Vietnam

## Abstract

Parkinson's disease (PD), which is characterized by the decreased motor function and the loss of dopaminergic neurons, is a common neurodegenerative disorder in elders. There have been numerous *in vitro* and *in vivo* models developed to study mechanisms of PD and screen potential drug. Recently, *dUCH*-knockdown *Drosophila* model has been established and showed potential for screening antioxidants for PD treatment. The dUCH-knockdown *Drosophila* model of PD mimics most of main PD pathologies such as dopaminergic neurons degeneration, locomotor dysfunction, and shortage of dopamine in the brain. Common purslane (*Portulaca oleracea* L.) is a nutritious vegetable containing a variety of antioxidants, levodopa, and dopamine, a neurotransmitter closely related to PD. Purslane has been reported to exert neuroprotective effects against several neurotoxins including rotenone and 6-OHDA in PD models. However, the recent data have not provided sufficient evidence for using purslane to treat PD or decelerate disease progression. Therefore, in this study, we utilized *dUCH*-knockdown fly to evaluate the capacity of purslane extracts for PD treatment. The results showed that purslane extracts improved locomotor ability in the larval stage and decelerated disease progression in the adult stage. Additionally, purslane extracts also reduced dopaminergic neuron degeneration. Taken together, our data strongly demonstrated that purslane extracts effectively rescued PD-like phenotypes in the fly model. This result contributed a foundation for further study on the application of purslane in PD treatment.

## 1. Introduction

Parkinson's disease (PD) is the second most common neurodegenerative disorder and affects approximately 3% of the population over the age of 65 [1]. The term parkinsonism refers to a clinical syndrome characterized by combinations of motor impairments including bradykinesia, tremor at rest, muscle rigidity, loss of postural reflexes, flexed posture, and freezing phenomenon [1]. Moreover, nonmotor symptoms such as fatigue, depression, anxiety, sleep disturbances, and dementia have been also reported in PD patients [1]. The neuropathological hallmarks of PD are the loss of neuromelanin-containing dopamine (DA) neurons of the substantia nigra and the appearance of Lewy body in the cytoplasm of cells [1]. In a view of genetics, many genes and their variants including *α-synuclein, LRRK2, PARKIN, DJ-1, PINK-1, GBA*, and *UCH-L1* have been demonstrated to be associated with PD [2, 3]. Besides that, the exposure to some neurotoxin such as MPTP, 6-OHDA, rotenone, and paraquat increases the risk of PD [4].

Many animal models which can simulate PD symptoms have been developed for not only researching mechanisms of PD but also screening medicines for treatments. Mouse is considered as an outstanding model of PD because of the high similarity between this organism and human. There have been many remarkable PD mouse models induced by neurotoxins (such as MPTP and paraquat) and genetic factors (such as *PARKIN, PINK-1, DJ-1, LRRK-2*, and *α-Synuclein*) [5]. However, the limitation of experimental population size is a disadvantage to applications of mouse models [6]. Therefore, small animal models such as *C. elegans*, *Drosophila*, and zebrafish have been used for high-throughput screening of potential drug candidates and studying pathogenic mechanisms [7–9]. Notably, three FDA-approved drugs for the treatment of Huntington's disease, another neurodegenerative disorder, were successfully identified using *Drosophila* model [8]. *Drosophila* models have been established by exposure to neurotoxins such as paraquat, rotenone, or genetic modifications or combinations of these factors [10]. In 2018, Tran et al. developed a novel PD fly model by knocking down *dUCH*, a homolog gene of human *UCH-L1*, in DA neuron. This fly model of PD exhibited PD-like phenotypes including defects in locomotor abilities and progressive DA neuron degeneration [11].

On the other hand, the high levels of reactive oxygen species (ROS), which mainly cause serious oxidative stress, were found in the substantia nigra of PD patients [12]. Oxidative stress can impair the structures of proteins, lipids, carbohydrates, and nucleic acids, resulting in cell damage [13]. In that circumstance, proteasome activity plays an important role in minimizing and blocking negative effects of ROS on cellular structures by removing damaged proteins, providing replaced substrates for oxidation and decaying faulty mitochondria [12]. In contrast, dysfunctions of the ubiquitin-proteasome system can dramatically increase oxidative stress and damage and reduce the mitochondrial function, leading to cell death [12].

Based on the link between oxidative stress and PD, antioxidant treatment has been considered as a potential therapy for PD [14, 15]. PD models which exhibit oxidative stress have been used for screening and evaluating potential antioxidants for treating PD [11]. Multiple antioxidants were reported that they could prevent and reduce disease progression in models of PD [16, 17]. Antioxidants such as Vitamin E, Vitamin C, and curcumin have been reported to mitigate the PD-like phenotype in fly models [18–20]. Notably, it was demonstrated that knockdown of *dUCH* led to increases in ROS levels, and antioxidants including vitamin C and curcumin could improve the PD-like phenotypes in this fly model [11, 21]. Therefore, *dUCH*-knockdown fly is a potential model for screening antioxidants which can be used for PD treatment.

Purslane (*Portulaca oleracea* L.) is a common vegetable and collected primarily for food and medicines. Purslane seed powder can be used as an ingredient in mush and bread. Fresh purslane is also commonly used in salads. Besides being consumed as food, purslane has been used as a folk medicine for treating fever, burn, headache, parasitic worms, and so forth in various countries [22]. Purslane is a good source of omega-3 fatty acid; vitamins A, B, and C; and minerals such as potassium, magnesium, calcium, phosphorus, and iron [23]. Many other compounds including flavonoids, alkaloids, terpenoids, polysaccharides, saponins, and tannins were isolated from purslane [22, 23]. These compounds are known to have anti-inflammatory, antibacterial, and antioxidant effects [23, 24]. Furthermore, purslane contains dopamine, a neurotransmitter closely related to PD, and levodopa, a precursor of dopamine [24]. Purslane was demonstrated to have protective effects in neurotoxin-induced models of PD. 6-OHDA-induced mice treated with purslane showed an improvement in motor ability after 15 days and a decrease in dopaminergic neuronal cell death after 4 weeks [25]. Oleracein E from purslane rescued rotenone-induced SH-SY5Y human neuroblastoma cells from apoptosis [26]. However, there has not been sufficient evidence for the ability of purslane to treat PD, especially in genetic models.

In this study, we utilized the *Drosophila* model of PD with *dUCH* knockdown in order to evaluate the ability of purslane extracts to treat PD.

## 2. Materials and Methods

### 2.1. Fly Strains and Maintenance

Fly stocks were cultured on standard food containing 5% dry yeast, 5% sucrose, 3% powdered milk, and 0.85% agar at 25°C. In the experiments, purslane extracts was added to standard food at suitable concentrations and then maintained in the dark to protect against the loss of antioxidant activity.

Wild-type strain Canton-S was obtained from the Bloomington *Drosophila* Stock Center (BDSC). RNAi line carrying *UAS-dUCH-IR* fusion (GD#26468) for knocking down *Drosophila Ubiquitin Carboxyl-terminal Hydrolase* (*dUCH*, CG4265) was received from the Vienna *Drosophila* Resource Center (VDRC). dsRNAs produced from the RNAi line are specific for *dUCH* in *Drosophila melanogaster*. RNAi line carrying *UAS-GFP-IR* for dsRNA control was obtained from BDSC (#56179). *TH-GAL4* (Tyrosine hydroxylase) driver (BDSC#8848) (*ple-GAL4*) was used to specifically knock down *dUCH* in dopaminergic neurons. Control flies were generated by crossing Canton-S or flies carrying *UAS-GFP-IR* with the *TH-GAL4* driver. All experiments using the GAL4/UAS system in the present study were performed at 28°C.

### 2.2. Herb

The purslane (*P. oleracea* L.), voucher specimen number PHH0000678, was harvested at Duc Trong vegetable garden, Lam Dong province, Viet Nam in February 2017.

### 2.3. Preparing the Aqueous Purslane Extract

The fresh purslane which included roots, stems, and leaves was harvested and then washed immediately after the roots were removed. After being drained, the sample was freeze-dried until completely dry. The dried purslane was ground into powder, and then stored at −30°C. The purslane powder was well mixed with water in a ratio 1 : 15 (weight: volume). The mixture was kept for 30 minutes at 4°C and then centrifuged at 7000g, 4°C for 20 minutes. After centrifugation, the supernatant solution was collected and frozen at −30°C and then completely dried by freeze-drying. The aqueous purslane extracts powder was stored at −30°C for the next experiments. In order to preserve antioxidant compounds, the purslane extracts and mediums containing the extract were limited exposure to light, heat, and air during the extracting process and all experiments in this research.

### 2.4. DPPH Free Radical Scavenging Assay

This assay is based on the discoloration of DPPH (1,1-diphenyl-2-picrylhydrazyl) (#D9132, Sigma, Singapore) when this chemical reacts with antioxidant compounds. The amount of remaining DPPH after the reaction is evaluated by measuring the absorbance at a wavelength of 517 nm. The DPPH assay followed the protocol of Sharma and Bhat [27] with some adjustments.

A reaction consisted of methanol solvent, 50 *μ*M DPPH, and samples depended by each experiment. In the experiment examining the antioxidant capacity of purslane extract, the extract added to the reaction at final concentrations of 25, 50, 100, and 200 *μ*g/mL. Vitamin C, L-Ascorbic acid (# A0278-25G, Sigma, Singapore) was used as a standard antioxidant substance. Vitamin C was added to the reaction at final concentrations of 5, 10, 15, 20, and 25 *μ*M. In the experiment evaluating the antioxidant accumulation in larvae, larval extract solutions were prepared by rubbing 50 larvae of each strain with 500 *μ*l of distilled water and the supernatant was collected after being centrifuged at 4°C for 20 minutes. The larval extract solutions were added to DPPH reaction at serial dilutions of 1/2, 1/4, 1/8, and 1/16. There were also the blanks for each sample with no DPPH and negative control. The reaction took place in a light-resistant condition for 40 minutes at 30°C. After the reaction, the amount of remaining DPPH was evaluated by measuring the absorbance (Abs) at 517 nm.

The antioxidant activity (AA) at each concentration was calculated by the formula [28]:

(1) $$\begin{array}{c} \text{\%AA}=100-\frac{\text{Abs}\;\;\text{sample}-\text{Abs}\;\;\text{blank}\;\;\text{ sample}}{\text{Abs }\;\;\text{negative}\;\;\text{ control}}*100. \end{array}$$

Graphs were drawn using GraphPad Prism 7.00 (GraphPad Software, USA) with accordant algorithms. The half maximal inhibitory concentration (IC_50_) was calculated based on the graph.

The relative accumulated antioxidant in extract-treated larvae compared with nontreated larvae was calculated by the formula:

(2) $$\begin{array}{c} y=\frac{\text{IC}50\text{ of nontreated larvae}}{\text{IC}50\text{ of treated larvae at each concentration}}-\frac{\text{IC}50\text{ of nontreated larvae}}{\text{IC}50\text{ of nontreated larvae}}. \end{array}$$

Raw data were collected by Microsoft Excel 2016 (Microsoft, USA). The average index was calculated and graphed using GraphPad Prism 7.00.

### 2.5. Growth Rate and Toxicity

We collected 70 embryos of each fly strains and transferred into a tube with extract-containing medium (3 tubes per concentration). The serial of purslane concentration used was 0; 1.25; 2.5; 5; 10; 15; 20; 25 mg/mL. The number of pupae formed and the number of flies emerged were recorded daily. The toxicity of purslane extracts was evaluated through calculating the percentage of adult flies which were still alive after one day at each concentration compared with that of untreated flies. Raw data were collected by Microsoft Excel 2016 (Microsoft, USA). The average index was calculated, statistically analyzed, and graphed using GraphPad Prism 7.00 (GraphPad Software, USA).

### 2.6. Feeding Assay

Feeding assay was performed to measure the amount of food eaten by larvae during a period of time. The food intake is quantified indirectly through the amount of a dye added to medium consumed by larvae. Coomassie Brilliant Blue G-250 (# 808274-10g, Biomedicals, USA) was added to the extract-containing standard medium at a concentration of 2%. Early third-instar larvae cultured in the extract-containing medium were collected and fed dye-containing food within 30 minutes. Next, we transfer larvae into tubes at a density of 10 larvae per tube, rubbed samples in PBS, and centrifuged. The supernatant was collected, 10% ethanol was added, and absorbance was measured at a wavelength of 595 nm. The relative antioxidant intake was estimated based on the amount of purslane extract-containing food consumed by larvae at each concentration compared with the lowest concentration of 1.25 mg/mL. Raw data were collected by Microsoft Excel 2016 (Microsoft, USA). The average index of each strain was calculated, statistically analyzed, and graphed using GraphPad Prism 7.00 (GraphPad Software, USA).

### 2.7. Crawling Assay

The locomotor ability in the larval stage can be evaluated by measuring the crawling speed, which could show the effects of purslane extracts on the early stage of the disease. Crawling assay was performed as described in a study of Nichols et al. [29] with some modifications. 30 male larvae in the third-instar stage were randomly chosen and placed on a 2% agar Petri dish at a density of three larvae. The movement of these larvae was recorded within 1 minute. These videos were analyzed using the ImageJ software (NIH, USA) and the wrMTrck plugin (developed by Dr. Jesper Søndergaard Pedersen, http://www.phage.dk/plugins/wrmtrck.html) for obtaining average speed data. Raw data were collected by Microsoft Excel 2016 (Microsoft, USA). The average speed of each strain was calculated, statistically analyzed, and graphed using GraphPad Prism 7.00 (GraphPad Software, USA).

### 2.8. Rapid Iterative Negative Geotaxis (RING) Assay (Climbing Assay)

The RING assay is based on gravity reversal reaction when there is a force acting in the same gravity direction on fly [29]. More healthy flies are able to climb higher in the same period of time. This experiment can be performed to test the effect of a factor on the mobility of adult fly. This assay was performed as described previously [30] with some modifications. After being fed extract-containing standard medium, newly eclosed flies were anesthetized with diethyl ether and randomly selected to collect 160–200 male flies. These flies were then divided into 2 groups including short-term treatment group and long-term treatment group. The short-term treatment group was fed on the standard medium, while the long-term treatment group continued to be fed the extract-containing standard medium. The flies were maintained at 28°C and transferred to tubes containing new medium every 2 days. The flies were kept on new medium overnight before carrying out the climbing assay. They were then anesthetized with diethyl ether and separated into climbing vials at a density of 10 flies per vial. These flies had at least 60 minutes of rest before the assay. After that, each vial was tapped to collect the flies to the bottom and the movement of these flies was recorded. This procedure was repeated 8 times. The climbing index was the average percentage of flies which passed the height of 6 cm after 5 seconds. The relative levels of improvements in climbing ability were calculated as follows: climbing index of 11-day-old flies divided by mean of climbing index of 1-day-old flies and then normalized by the mean of nontreated control. Raw data were collected by Microsoft Excel 2016 (Microsoft, USA). The value was calculated, statistically analyzed, and graphed using GraphPad Prism 7.00 (GraphPad Software, USA).

### 2.9. Immunostaining, Imaging, and DA Neuron Quantification

Immunofluorescence was performed following a standard protocol [31] with some modifications. Tyrosine hydroxylase (TH) enzyme, which catalyzes a conversion of L-tyrosine to L-DOPA, a precursor of dopamine, was used as a marker for DA neurons. Larval and adult brains were dissected in cold phosphate-buffered saline (PBS) and fixed in 4% paraformaldehyde at 25°C for 22 minutes. After being washed with 0.3% PBS-T (PBS containing 0.3% Triton-X100) twice, the brains were blocked with blocking solution (0.15% PBS-T containing 10% normal goat serum) at 25°C for 30 minutes. Brains were then incubated with a primary antibody and rabbit anti-Tyrosine Hydroxylase (anti-TH; Millipore, AB152, Japan) and diluted in blocking solution (1 : 250) at 4°C for 36 hours. After being washed with 0.3% PBS-T, brains were incubated with secondary antibodies conjugated with Alexa 594 (1 : 500; Invitrogen) at 25°C for 2 hours, washed, and mounted in Vectashield Mounting Medium (Vector Laboratories, Japan). Samples were observed using Nikon fluorescence microscopy Ni-U (Nikon, Japan). The number of DA neurons in each cluster was manually counted using the cell counter plugin of ImageJ 1.49o (NIH, USA). Raw data were collected by Microsoft Excel 2016 (Microsoft, USA). The average number of DA neurons was calculated, statistically analyzed, and graphed using GraphPad Prism 7.00 (GraphPad Software, USA).

## 3. Results

### 3.1. Antioxidant Capacity of *P. oleracea* L. Extract

**Figure 1:**
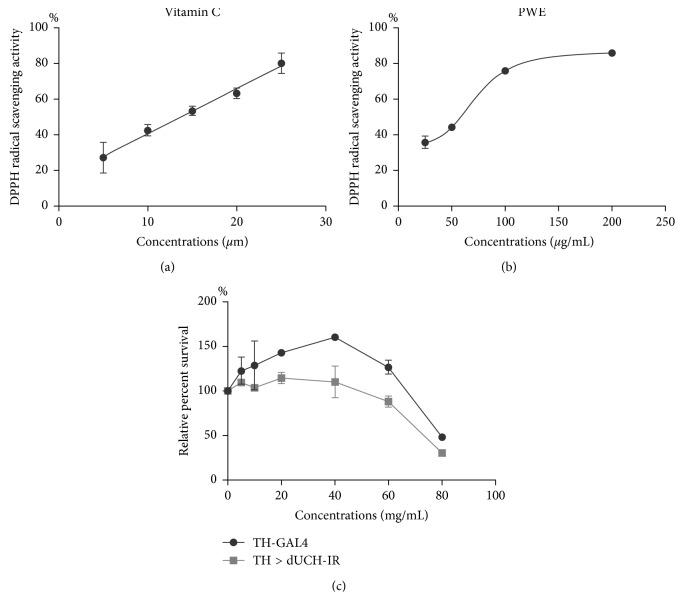
The antioxidant capacity and toxicity of *P. oleracea* L. extract. (a, b) The antioxidant capacities of PWE and vitamin C were evaluated by DPPH assay. (a) Vitamin C, *R* ^2^ = 0.9915, IC_50_ = 13.67 ± 2.03 *μ*M; (b) PWE, *R* ^2^ = 0.994, IC_50_ = 72.56 ± 3.03 *μ*g/mL (*n* = 3 and error bars represent the standard deviation of data); (c) effects of the purslane extracts on survival rate of one-day-old adult fly. Control line TH-GAL4 (*w/y*; +; *TH-GAL4*/*+*) and *dUCH*-knockdown line TH > dUCH-IR (*w/y*; +; *TH-GAL4/UAS-dUCH-IR*). Relative percent survival was calculated as follows: number of adult flies at each concentration of purslane extracts divided by number of nontreated adult flies (population size *N* = 70 and biological replication *n* = 2; error bars represent the standard deviation of data).

Purslane is known as a vegetable which has a high nutritional and antioxidant value [23]. In this study, to evaluate antioxidant capacity of *P. oleracea* L. aqueous extract (PWE), we performed the DPPH assay and determined the concentrations of PWE and vitamin C at which 50% of radical DPPH was inhibited (IC_50_ value) under determined conditions. The results showed that the IC_50_ value of PWE was 72.56 ± 3.03 *μ*g/mL which is equivalent to the antioxidant capacity of 13.67 ± 2.03 *μ*M vitamin C (Figures 1(a) and 1(b)). Vitamin C equivalent antioxidant capacities (VCEAC) of the PWE at different concentrations are shown in Supplementary Table S1.

### 3.2. Toxicity of *P. oleracea* L. Extract on *D. melanogaster*

In therapeutic drug discovery research, testing the toxicity of compounds or herbs is necessary to select suitable concentrations for further investigations [8]. We, therefore, evaluated the toxicity of PWE on the viability of fruit flies by measuring the percentage of embryos which could develop to one-day-old adult flies when treated with medium containing 0–80 mg/mL of the extract. The results showed that PWE at concentrations of less than 40 mg/mL increased the survival rate of *dUCH*-knockdown (TH > dUCH-IR) and control flies (TH-GAL4). At the concentration of 40 mg/mL, the survival rate of control and knockdown flies increased by 1.5 folds and 1.15 folds, respectively, compared with that of untreated flies. However, the survival rates of both *dUCH*-knockdown and control flies were significantly reduced when they were fed PWE at concentrations of 60 and 80 mg/mL (Figure 1(c)). The median lethal doses (LD_50_) of the purslane extracts in the control and *dUCH*-knockdown groups were 79.5 mg/mL and 73.2 mg/mL, respectively.

### 3.3. Food and Antioxidant Intake Abilities of Larvae Treated with *P. oleracea* L. Extract

**Figure 2:**
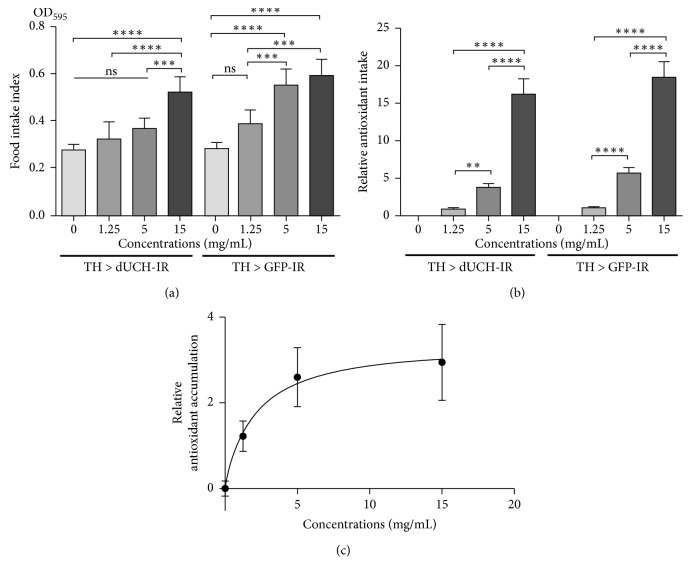
Food intake and antioxidant accumulating abilities of larvae when treated with *P. oleracea* L. extract. (a) Effects of PWE on the eating ability of third-instar larvae. The control line TH > GFP-IR (*w/y*; +; *TH-GAL4/UAS-GFP-IR*) and the *dUCH*-knockdown line TH > dUCH-IR (*w/y*; +; *TH-GAL4/UAS-dUCH-IR*). Population size *N* = 10 and biological replication *n* = 5; one-way ANOVA, post hoc test: Tukey, ns: not significant, ^*∗∗∗*^ *p* < 0.001, and ^*∗∗∗∗*^ *p* < 0.0001. Error bars represent the standard deviation of data. (b) The relative antioxidant intake based on the amount of food consumed by larvae. Control line (*+*; +; *TH-GAL4/UAS-GFP-IR*) and *dUCH*-knockdown line (+; +; *TH-GAL4/UAS-dUCH-IR*). Population size *N* = 10 and biological replication *n* = 5; one-way ANOVA, post hoc test: Tukey, ^*∗∗*^ *p* < 0.01, and ^*∗∗∗∗*^ *p* < 0.0001. Error bars represent the standard deviation of data. (c) The relative level of antioxidant accumulation by *dUCH*-knockdown larvae (*R* ^2^ = 0.9932, dissociation constant: *K*_d_ = 2.001 ± 0.437). Error bars indicate the standard error of the mean.

The PWE might have positive or negative impacts on eating ability of larvae, thereby affecting the amount of accumulated antioxidant. Thus, we performed experiments in which the third-instar larvae were treated with serial concentrations of PWE including 0, 1.25, 5, and 15 mg/mL and evaluated their food and antioxidant intake abilities. There were increases in larval eating ability in both *dUCH*-knockdown and control lines when treated with PWE. At the concentration of 15 mg/mL, the amounts of food which were ingested by treated knockdown and control larvae increased significantly by 1.9 and 2.1 folds, respectively, compared with untreated flies (Figure 2(a)). Consequently, the antioxidant intakes were increased by 4.5 and 19.3 folds when the knockdown flies were treated with 5 and 15 mg/mL PWE, respectively, compared with when knockdown flies were treated with 1.25 mg/mL PWE (Figure 2(b)).

On the other hand, the antioxidant accumulation of the treated knockdown larvae was also investigated through measuring antioxidant capacity of larval extract. The results showed that the antioxidant capacity of knockdown larvae extract which were treated with 1.25 mg/mL PWE increased by 2.2 folds compared with that of untreated knockdown larvae (Table 1). The levels of antioxidants in *dUCH*-knockdown larvae treated with 5 mg/mL or 15 mg/mL of PWE were 3.6-fold and 3.9-fold, respectively, higher than that of untreated knockdown larvae (Table 1). Interestingly, the relative level of accumulated antioxidant in the treated knockdown larvae which was estimated based on the antioxidant accumulation of larvae was rapidly increased by 1.63 folds when the PWE concentration increased from 1.25 mg/mL to 5 mg/mL (Figure 2(c)). However, the rate of increase in accumulated antioxidant slowed down, rising by only 1.08 folds, when the PWE concentration increased from 5 mg/mL to 15 mg/mL (Figure 2(c)). These results suggested that absorbed antioxidant was almost saturated at 5 mg/mL PWE.

### 3.4. *P. oleracea* L. Extract Rescued Locomotor Dysfunction Caused by Knockdown of *dUCH*

Previous study showed that DA neuron-specific *dUCH*-knockdown flies exhibited locomotor dysfunction in both larval and adult stages. Vitamin C treatment at 0.5 mM could improve locomotor ability of *dUCH-*knockdown larvae [11]. The above-mentioned results demonstrated that PWE also has antioxidant activity similar to vitamin C. We, therefore, investigated the effects of PWE on the locomotive ability of larvae by crawling assay and adult flies by climbing assay.

**Figure 3:**
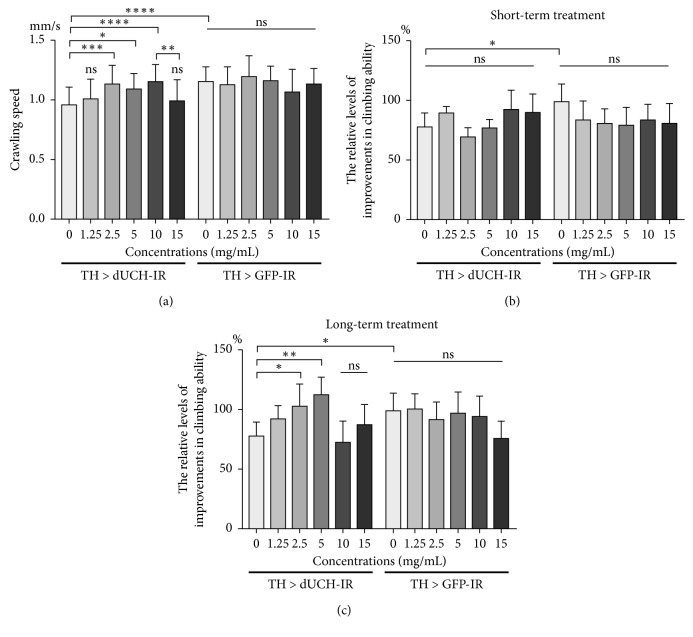
*P. oleracea* L. extract rescued locomotor dysfunction caused by knockdown of *dUCH.* (a) Effects of PWE on motion ability of third-instar larvae. Control line TH > GFP-IR (*w/y*; +; *TH-GAL4/UAS-GFP-IR*) and *dUCH*-knockdown line TH > dUCH-IR (*w/y*; +; *TH-GAL4/UAS-dUCH-IR*). *n* = 40; one-way ANOVA, post hoc test: Tukey, ns: not significant, ^*∗*^ *p* < 0.05, ^*∗∗*^ *p* < 0.01, ^*∗∗∗*^ *p* < 0.001, and ^*∗∗∗∗*^ *p* < 0.0001. Error bars represent the standard deviation of data. (b) Effects of short-term treatment with PWE on climbing ability of adult flies. Short-term treatment: the treatment was interrupted after the third-instar larval stage. Control line (*w/y*; +; *TH-GAL4/UAS-GFP-IR*) and *dUCH*-knockdown line (*w/y*; +; *TH-GAL4/UAS-dUCH-IR*). Population size *N* = 10 and biological replication *n* = 6; one-way ANOVA, post hoc test: Dunnett, ns: not significant, and ^*∗*^ *p* < 0.05. Error bars represent the standard deviation of data. (c) Effects of long-term treatment with PWE on climbing ability of adult flies. Long-term treatment: consecutively treating from embryos to 11-day-old flies. Control line (*w/y*; +; *TH-GAL4/UAS-GFP-IR*) and *dUCH*-knockdown line (*w/y*; +; *TH-GAL4/UAS-dUCH-IR*). Population size *N* = 10 and biological replication *n* = 6; one-way ANOVA, post hoc test: Dunnett, ns: not significant, ^*∗*^ *p* < 0.05, and ^*∗∗*^ *p* < 0.01. Error bars represent the standard deviation of data.

In the larval stage, we found that mobility of knockdown larvae (TH > dUCH-IR) increased after being treated with 2.5, 5, and 10 mg/mL of PWE. The crawling speeds of *dUCH*-knockdown larvae treated with 2.5, 5, and 10 mg/mL of PWE were higher (1.133, 1.092, and 1.153 mm/s, respectively) than that of untreated knockdown larvae (0.958 mm/s) (Figure 3(a)). Interestingly, crawling ability of control larvae (TH > GFP-IR) was not influenced by PWE treatment (Figure 3(a)). However, there was a decrease in crawling ability of *dUCH-*knockdown larvae after being treated with PWE at 15 mg/mL compared with that of knockdown larvae treated with 10 mg/mL of the extract (Figure 3(a)). This might be due to the side effects of purslane extracts at a high concentration on fly physiology, which might, in turn, affect the mobility of larvae.

In the adult fly, PWE treatment resulted in improvements in climbing ability of *dUCH*-knockdown flies when these flies were continuously treated with the extract at doses of 2.5 mg/mL and 5 mg/mL (Figure 3(c)). However, when knockdown flies were treated with PWE from the first instar stage and the treatment stopped at the pupa stage, the locomotion dysfunction did not improve in the adult flies (Figure 3(b)). This suggested that the progressive locomotive defects in *dUCH*-knockdown flies could be improved by long-term treatment with PWE at suitable concentrations.

### 3.5. *P. oleracea* L. Extract Rescued Degeneration of Dopaminergic Neurons Caused by Knockdown of *dUCH*

**Figure 4:**
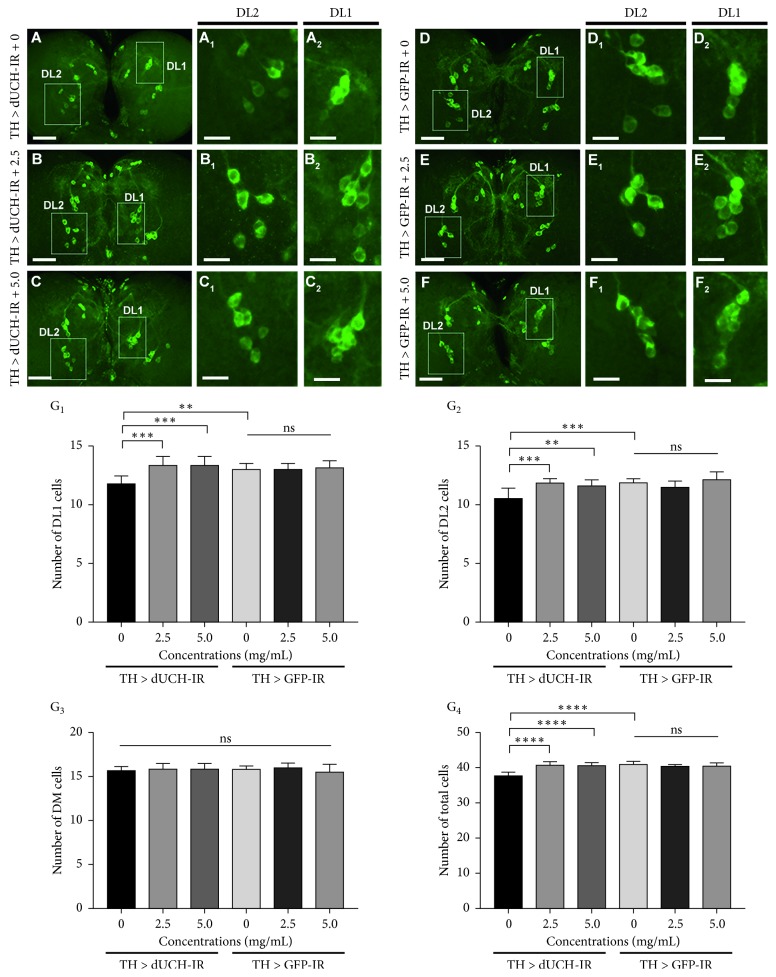
*P. oleracea* L. extract rescued degeneration of dopaminergic neurons caused by knockdown of *dUCH* in larvae. (A–F) Immunostaining images of brain lobes with antityrosine hydroxylase antibody, a marker for dopaminergic neurons. The control TH > GFP-IR (*w/y*; +; *TH-GAL4/UAS-GFP-IR*) and *dUCH-*knockdown larvae TH > dUCH-IR (*w/y*; +; *TH-GAL4/UAS-dUCH-IR*) were treated with 0, 2.5, and 5 mg/mL of PWE. Scale bars indicate 50 *μ*m. Dopaminergic neurons in DL2 and DL1 clusters are shown in (A_1_)–(F_1_) and (A_2_)–(F_2_), respectively, and scale bars indicate 20 *μ*m. (G_1–4_) The quantified data of the numbers of dopaminergic neurons in three clusters ((G_1_): DL1, (G_2_): DL2, (G_3_): DM, (G_4_): sum of three clusters including DL1, DL2, and DM). *n* = 8; one-way ANOVA, post hoc test: Tukey, ^*∗∗*^ *p* < 0.01, ^*∗∗∗*^ *p* < 0.001, and ^*∗∗∗∗*^ *p* < 0.0001. Error bars represent the standard deviation of data.

In Parkinson's disease, locomotor defects are attributed to the loss of DA neurons in the brain, and previous study showed that DA neuron-specific knockdown of *dUCH* caused DA neuron degeneration and the DA neuron degeneration is randomly occurred in different clusters [11]. In this study, the results of mobility assays showed that purslane extracts at the concentrations of 2.5 and 5 mg/mL could improve the crawling ability of *dUCH*-knockdown larvae (Figure 3(a)). Thus, we continued to investigate the effects of purslane extracts on the degeneration of DA neurons in the *dUCH*-knockdown larvae (TH > dUCH-IR) and the control larvae (TH > GFP-IR) at the concentrations of 0, 2.5, and 5 mg/mL by immunostaining with anti-TH antibody, a marker for DA neurons. We also found that knockdown of *dUCH* led to decreases in numbers of neurons in the DL1 (DL-dorsal lateral) and DL2 clusters and the total number of DA neurons in three clusters including DM (DM-dorsal medial), DL1, and DL2 (Figures 4(A), 4(D), and 4(G)) (decrease 1-2, 1, and 3 cells, respectively). In the DM cluster, there was no significant difference in numbers of DA neurons between control and knockdown flies (Figure 4(G3)). PWE did not affect the numbers of DA neurons in control larvae at both concentrations of 2.5 and 5 mg/mL (Figures 4(D), 4(E), 4(F), and 4(G)). As expected, in the *dUCH*-knockdown larvae, 2.5 and 5 mg/mL of the extract could prevent against neurodegeneration in both DL1 and DL2 clusters (Figures 4(A), 4(B), 4(C), and 4(G_1-2_)), as well as the total of three clusters (Figure 4(G4)).

**Figure 5:**
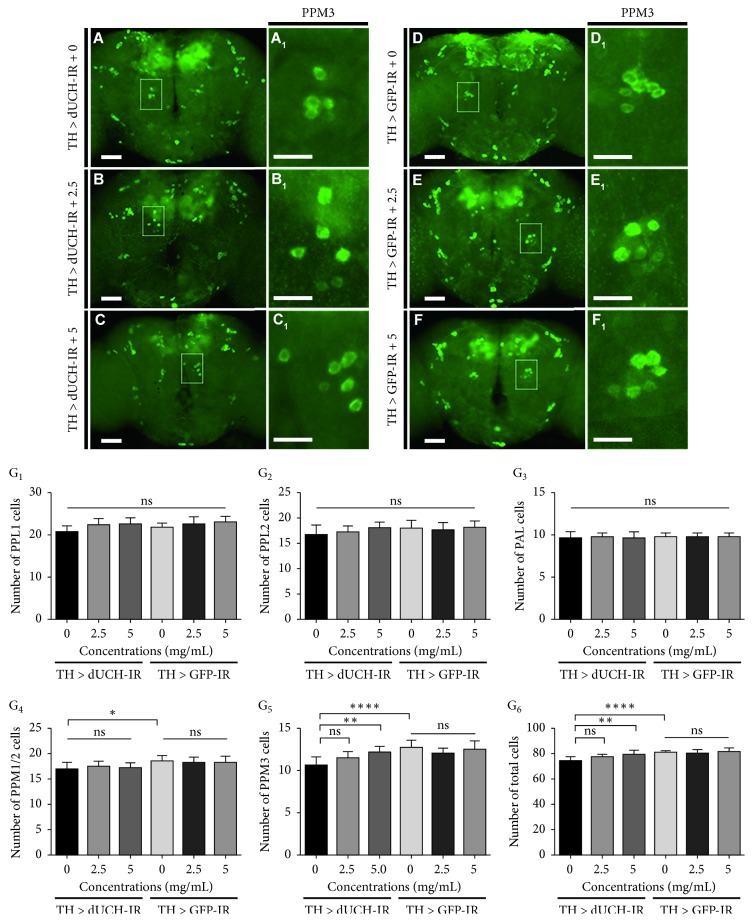
*P. oleracea* L. extract rescued degeneration of dopaminergic neurons caused by knockdown of *dUCH* in adult flies. (A–F) Immunostaining images of the adult brains with antityrosine hydroxylase antibody, a marker for dopaminergic neurons. The control (TH > GFP-IR) and *dUCH*-knockdown larvae (TH > dUCH-IR) were treated with 0, 2.5, and 5 mg/mL of PWE. Scale bars indicate 50 *μ*m. (A_1_)–(F_1_) Dopaminergic neuron cluster PPM3, scale bars indicate 20 *μ*m. (G_1–6_) The quantified data of the numbers of dopaminergic neurons in five clusters (G_1_): PPL1, (G_2_): PPL2, (G_3_): PAL, (G_4_): PPM1/2, (G_5_): PPM3, (G_6_): Sum of five clusters). *n* = 8; one-way ANOVA, post hoc test: Tukey, ns: not significant, ^*∗*^ *p* < 0.05, ^*∗∗*^ *p* < 0.01, and ^*∗∗∗∗*^ *p* < 0.0001. Error bars represent the standard deviation of data.

Furthermore, the loss of DA neurons caused by knockdown of *dUCH* was also reduced by PWE treatment in adult fly. In *dUCH*-knockdown untreated flies, the numbers of DA neurons in PPM1/2 and PPM3 clusters and the total of five clusters (PPL1, PPL2, PPM1/2, PPM3, and PAL) were lower than those of control flies (Figures 5(A), 5(D), and 5(G_4,5,6_)). When the *dUCH*-knockdown flies were treated with 5 mg/mL PWE, there were improvements in the number of DA neurons in PPM3 cluster and the total of five clusters compared with the untreated flies (Figures 5(A), 5(C), and, 5(G_5,6_)). However, there were not significant improvements in the numbers of DA neuron in *dUCH*-knockdown flies treated with 2.5 mg/mL PWE (Figures 5(A), 5(B), and 5(G)). The purslane extracts did not affect the numbers of DA neurons in the control flies at both concentrations of 2.5 and 5 mg/mL (Figures 5(D)–5(G)). These results suggested that PWE at suitable concentrations could decrease the degeneration of DA neurons which might lead to the improvements in locomotive ability of *dUCH*-knockdown flies.

### 3.6. Effects of P. oleracea L. Extract on *D. melanogaster* Development

**Figure 6:**
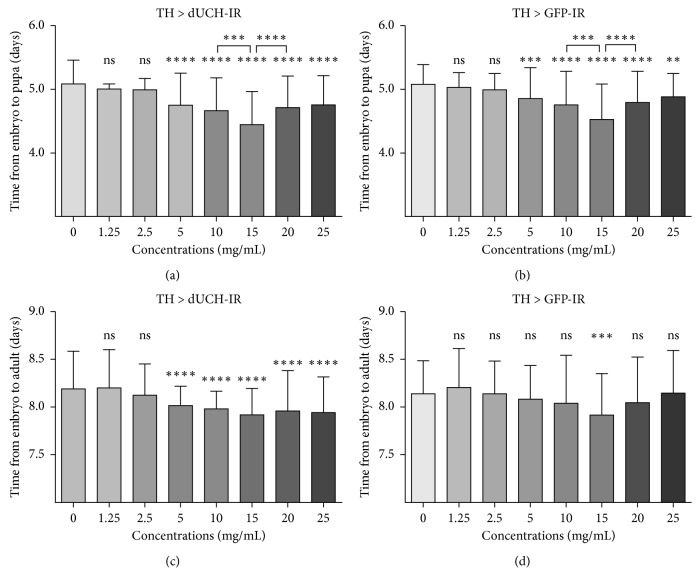
Effects of *P. oleracea* L. extract on *D. melanogaster* development. Control line (TH > GFP-IR) and *dUCH-*knockdown line (TH > dUCH-IR). (a, b) Period from embryo stage to pupa stage. (c, d) Period from embryo stage to adult stage. (*n*_a_ = 145, *n*_b_ = 133, *n*_c_ = 145, and *n*_d_ = 131; one-way ANOVA, post hoc test: Tukey, ns: not significant, ^*∗∗*^ *p* < 0.01, ^*∗∗∗*^ *p* < 0.001, and ^*∗∗∗∗*^ *p* < 0.0001). Error bars represent the standard deviation of data.

To address if PWE has any effect on *Drosophila melanogaster* development, we carried out experiments in which flies were treated with the extract at different doses and different stages of development. In the stage from embryo to pupa, our results showed that there were no significant differences in growth time between treated and untreated flies when control (TH > GFP-IR) and *dUCH*-knockdown (TH > dUCH-IR) flies were treated with PWE at doses less than 2.5 mg/mL. However, the extract at concentrations of 5 to 25 mg/mL reduced the growth time of treated flies compared with that of untreated flies in both control and knockdown flies (Figures 6(a) and 6(b)). In the stage from embryo to adult fly, only the control flies treated with 15 mg/mL of the extract exhibited a decline in developmental time compared with untreated control flies (Figure 6(c)). The purslane extracts at 5 to 25 mg/mL shortened growth time of *dUCH*-knockdown fly compared with that of untreated knockdown flies, and this was similar to the effects of PWE in the stage from embryo to pupa (Figures 6(a) and 6(c)). Notably, in both stages, growth time tended to decrease at concentrations of 0 to 15 mg/mL and then increase at higher concentrations (Figures 6(a)–6(d)) in both control and knockdown flies.

## 4. Discussion

*P. oleracea* L. is known as a vegetable which contains a high level of nutrients, especially antioxidants [23]. Previous studies showed that purslane could improve disease symptoms caused by oxidative stress [32]. Treating high-fat mice with purslane extracts reduced lipid peroxidation and increased activities of antioxidant enzymes in blood and liver [32]. Besides that, purslane exerted neuroprotective effects against some neurotoxins in 6-OHDA-induced rat model and rotenone-induced cellular and mouse models of PD [25]. In this study, purslane aqueous extract showed its high potential for PD treatment by improvements in PD-like phenotypes in the fly model with *dUCH* knockdown.

It is well known that the basic symptoms of Parkinson's disease are difficulty in walking, slow movement, and tremor [33]. Therefore, the model for studying PD should display pathophysiologic features and symptoms of PD. Previous study revealed that *D. melanogaster* model with knockdown of *dUCH*, a homolog of human *UCH-L1*, could mimic PD symptoms; therefore, this is a potential tool for drug screening [11]. DA neuron-specific knockdown of *dUCH* caused long-term adverse effects on locomotor abilities in both larval and adult stages. However, the crawling ability of *dUCH*-knockdown larvae improved when being treated with PWE. Similarly, PWE treatment could slow down the decrease in the climbing ability of *dUCH*-knockdown flies. Furthermore, we also found that DA neuron degeneration was decreased by PWE treatment in both larval and adult stages. Therefore, we suggest that purslane treatment might protect against locomotor defects and reduction in the number of DA neurons caused by knockdown of *dUCH*. These results are similar to that given by curcumin treatment in this PD fly model [21]. It is known that high levels of reactive oxygen species (ROS) were found in the substantia nigra of PD patients and antioxidant treatment has been considered as a potential therapy for PD [14, 15]. Our previous study also demonstrated that an increased level of ROS was involved in the induction of PD-like phenotypes, while curcumin, an antioxidant, rescued those phenotypes in this fly model [11, 21]. Our results showed that the half maximal inhibitory concentration (IC_50_) of PWE for scavenging DPPH free radicals was 72.56 ± 3.03 *μ*g/mL, and that of ascorbic acid was 19.48 *μ*M. That means the antioxidant activity of 100g purslane extracts was equivalent to that of 473 ± 14.32 mg Vitamin C. The results provided a good explanation for purslane activity on PD treatment.

On the other hand, purslane was reported to have weak or no cytotoxic activities in *in vitro* models. The LD_50_ of methanolic extract in acute toxicity experiment on mice was 1853 mg/kg, suggesting that purslane is a moderately toxic plant according to WHO classification [34]. In addition, the clinical trial of purslane at dose 180 mg/day for 12 weeks in type 2 diabetes patients showed that purslane consumption is safe with no significant differences in hematologic, biochemistry, and urinalysis values between the purslane-treated and placebo groups [35]. In our research, the LD_50_ of purslane on the *dUCH*-knockdown *Drosophila* was 73.2 mg/mL. The LD_50_ concentration was 14 folds higher than the effective concentration (5 mg/mL) for PD treatment on this fly model. However, it should be noted that from the concentration of 40 mg/mL, purslane extract started to express negative impact on *Drosophila* vitality. The side effects might be due to the excessive antioxidant amount which accumulated when the flies were feed on medium containing high concentration of purslane extract. This hypothesis is correlative to previous reports on drawbacks of high-dose antioxidant. Besides, the toxicity of purslane extract can be caused by accumulating large amounts of the extract's components such as heavy metals [36].

In addition, the ability to absorb food also affects the effectiveness of the treatment by oral route and interferes with the results of the experiment. For example, the ingestion of food without digestion or absorption can lead to imprecise evaluation of the effective concentration. Therefore, food and nutrient intake is the essential assessment to properly evaluate the effect of potential compounds on the disease model. Our study showed that despite the significant increases in both the food intake and the absorbed antioxidant at the concentrations of over 5 mg/mL, the increase in antioxidant accumulation slowed down. The results were similar to that of previous research in which antioxidant capacity in human plasma was increased to a limited level in the long-term high-antioxidant diet [37]. These data suggested that it might not be necessary to consume a high dose of antioxidant to avoid susceptible drawbacks.

In addition to the *dUCH*-knockdown model, there are many models of Parkinson's disease based on other pathogenic pathways or processes that are of interest [38]. Since dUCH-knockdown *Drosophila* model was proved to have a close link to oxidative stress, other *Drosophila* models which related to mitochondrial damage and oxidative stress such as the PINK-1 and PARKIN mutation and the overexpressing of *α*-SYNUCLEIN are suggested to be the next potential models to provide more evidence about the activity and usability of purslane for Parkinson's disease [10, 39, 40].

In conclusion, these results taken together demonstrated that *Portulaca oleracea* L. is a potential candidate for treating PD or supporting to control PD symptoms. The appropriate concentrations of purslane extracts at which PD-like phenotypes induced by knockdown of *dUCH* were improved and the negative impacts on fly physiology were limited were 2.5 mg/mL and 5 mg/mL.

## Figures and Tables

**Table 1:** The IC_50_ of *dUCH*-knockdown larval extract in DPPH assay^*∗*^; flies were treated with the concentrations of 0–15 mg/mL PWE.

Fly lines	IC_50_	Standard error
*dUCH*-Kd	0.9404	0.0827
*dUCH*-Kd + 1.25 mg/mL	0.4226	0.0292
*dUCH*-Kd + 5 mg/mL	0.2613	0.0269
*dUCH*-Kd + 15 mg/mL	0.2383	0.0325

^*∗*^Calculated by the dilution rate of larval extract at which the larval extract can convert 50% of DPPH radicals (population size *N* = 50). *dUCH*-Kd: *dUCH*-knockdown flies.

## Data Availability

The data used to support the findings of this study are available from the corresponding author upon request.

## Supplementary Materials

Supplementary Materials Table S1. Vitamin C equivalent antioxidant capacity (VCEAC) of the purslane extract.
