# Supplementary material for: Evaluating the Potential of Portulaca oleracea L. for Parkinson's Disease Treatment Using a Drosophila Model with dUCH-Knockdown
Source: Parkinsons Dis. 2019 Apr 18;2019:1818259. doi: 10.1155/2019/1818259 (PMC6501175; doi:10.1155/2019/1818259)
Supplement: Supplementary Materials — Table S1. Vitamin C equivalent antioxidant capacity (VCEAC) of the purslane extract. [file 1818259.f1.pdf]

Supplementary Materials

Table S1: Vitamin C equivalent antioxidant capacity (VCEAC) of the purslane extract

Purslane extract (mg/mL)	Vitamin C (mM) (mean ± SE)
1.25	0.24 ± 0.04
2.5	0.47 ± 0.09
5	0.94 ± 0.18
10	1.88 ± 0.36
15	2.83 ± 0.54
